# Integrating Multiple Interaction Networks for Gene Function Inference

**DOI:** 10.3390/molecules24010030

**Published:** 2018-12-21

**Authors:** Jingpu Zhang, Lei Deng

**Affiliations:** 1 School of Computer and Data Science, Henan University of Urban Construction, Pingdingshan 467000, China; zhangjp@csu.edu.cn; 2 School of Software, Central South University, Changsha 410075, China

**Keywords:** multiple interaction networks, function prediction, multinetwork integration, low-dimensional representation

## Abstract

In the past few decades, the number and variety of genomic and proteomic data available have increased dramatically. Molecular or functional interaction networks are usually constructed according to high-throughput data and the topological structure of these interaction networks provide a wealth of information for inferring the function of genes or proteins. It is a widely used way to mine functional information of genes or proteins by analyzing the association networks. However, it remains still an urgent but unresolved challenge how to combine multiple heterogeneous networks to achieve more accurate predictions. In this paper, we present a method named ReprsentConcat to improve function inference by integrating multiple interaction networks. The low-dimensional representation of each node in each network is extracted, then these representations from multiple networks are concatenated and fed to gcForest, which augment feature vectors by cascading and automatically determines the number of cascade levels. We experimentally compare ReprsentConcat with a state-of-the-art method, showing that it achieves competitive results on the datasets of yeast and human. Moreover, it is robust to the hyperparameters including the number of dimensions.

## 1. Introduction

With the advent of high-throughput experimental techniques, genome-scale interaction networks have become an indispensable way to carry relevant information [1,2,3,4,5]. Researchers can extract functional information of genes and proteins by mining the networks [6,7]. These methods are based on the fact that proteins (or genes) that are colocated or have similar topological structures in the interaction network are more likely to be functionally related [8,9,10,11,12,13,14,15,16,17,18]. Thus, we are able to infer the unknown characteristics of proteins based on the knowledge of known genes and proteins.

An important challenge to the methods of network based prediction is how to integrate multiple interaction networks constructed according to heterogeneous information sources (for example, physical binding, gene interactions, co-expression, coevolution, etc.). The existing methods of integrating multiple networks for functional prediction mainly combine multiple networks into a representative network, and then perform prediction algorithms [19] (for example, label propagation algorithm [20] and graph clustering algorithm [21]) on the integrated network. There are two main methods for integrating the edges of different networks: one is the weighted averaging method of edge weights [12,22] with GeneMANIA [23] as a representative. In GeneMANIA, the weight of each network is obtained by optimizing according to the functional category. The other is a method based on Bayesian inference [24,25], which is used to combine multiple networks into the protein interaction network in database STRING [26]. A key drawback of these methods of projecting various data sets into a single network representation is that the projection process can result in a large loss of information. For example, a particular context interaction pattern that exists only in a particular data sets (e.g., tissue-specific gene modules) is likely to be obscured by the edges from other data sources in the integrated network. Recently, Cho et al. proposed a new integration method, Mashup [27], which integrates multiple networks by compressing representations of topological relationships between nodes. Vladimir and the coauthors [28] developed deepNF to derive functional labels of proteins using deep neural networks for calculating network embeddings. The method could explore underlying structure of networks and showed improved performance. However, tuning the hyperparameters requires efforts and expertise.

In this paper, we propose a multinetwork integration method, ReprsentConcat, based on gcForest [29], which builds a deep forest ensemble with a cascade structure. The cascade structure enables gcForest to learn representations. Moreover, by multigrained scanning of high-dimensional input data, gcForest can further enhance the learning ability of representation and learn the context or structure information of features. In gcForest, the number of cascade levels can be automatically determined, improving the effect of classification. In ReprsentConcat, first, a feature representation of each node in the network is obtained according to the topological structure of one network, and these features could represent the intrinsic topology of the network. Secondly, considering that the high-dimensional features contain noise, we compact these features to obtain the low dimensional representations which explain the connectivity patterns in the networks. Finally, the features of the nodes in each network are concatenated to train the classifier as the input of gcForest. A 5-fold cross-validation experiment is performed on the networks including six protein interaction networks, and the experimental results show that ReprsentConcat outperforms state-of-the-art Mashup.

## 2. Results

### 2.1. Experimental Data Set

In order to verify the effectiveness of our proposed multinetwork integration algorithm, the function prediction of proteins is performed on multiple networks consisting of six protein–protein interaction networks. The six protein interaction networks and the annotations of proteins are derived from the work of Cho et al [27]. The raw datasets are available online at http://denglab.org/ReprsentConcat. In the dataset, protein interaction networks include species such as humans and yeast and so on, from the STRING database v9.1 [26]. Moreover, the networks constructed from text mining of the academic literature are excluded. As a result, the six yeast heterogeneous networks include a total of 6400 proteins, and the number of edges in these networks ranges from 1361 to 314,013 (as shown in Table 1). The six human heterogeneous networks include 18,362 proteins, and the number of edges in the networks ranged from 1880 to 788,166 (as shown in Table 1). The weights of edges in these networks are between 0 and 1, representing the confidence of the interaction.

The functional annotations for yeast proteins comes from Munich Information Center for Protein Sequences (MIPS) [30], and the annotations for human from the Gene Ontology (GO) database [31]. The functions in MIPS are organized in a tree structure and are divided into three levels, where Level 1 includes 17 most general functional categories, Level 2 includes 74 functional categories, and Level 3 includes 154 most specific functional categories. It is noted that each protein can have more than one function. The GO terms in the GO database are organized in a directed acyclic graph. The GO terms are divided into three categories including biological process (BP), molecular function (MF), and cellular component (CC), representing three different functional categories. In this dataset, these GO terms are divided into three groups where each consists of GO terms with 11–30, 31–100, and 101–300 annotated genes (see Table 2). In order to maintain the consistency of the predicted GO labels, the GO label is propagated in the GO hierarchy by applying the “is a” and “part of” relationships, i.e., if a gene is labeled as a GO term, then the gene is also annotated with all the ancestral terms of the term. 

### 2.2. Evaluation Metrics

In our ReprsentConcat, the output for each class is a real number between 0 and 1, and we obtain the final predictions by applying an appropriate threshold, *t*, on the outputs. For a given sample, if the corresponding output for a class is equal to or greater than the threshold *t*, this class is assigned to the sample; otherwise it is not assigned to the sample. However, choosing the “optimal” threshold is a difficult task. Low thresholds will bring about more classes being assigned to the sample, resulting in high recall and low precision. On the contrary, a larger threshold allows fewer classes to be assigned to the sample, resulting in high precision and low recall. To tackle this problem, we use Precision–Recall (PR-curve) as an evaluation metric. In order to plot the PR-curve of a given classifier, different thresholds in [0, 1] are respectively applied to the output of the classifier, so as to obtain the corresponding precision and recall. The area under the PR-curve (AUPR) can also be calculated, and different methods can be compared based on their area under the PR-curve.

### 2.3. Impact of Feature Dimension on Performance

**Figure 1:**
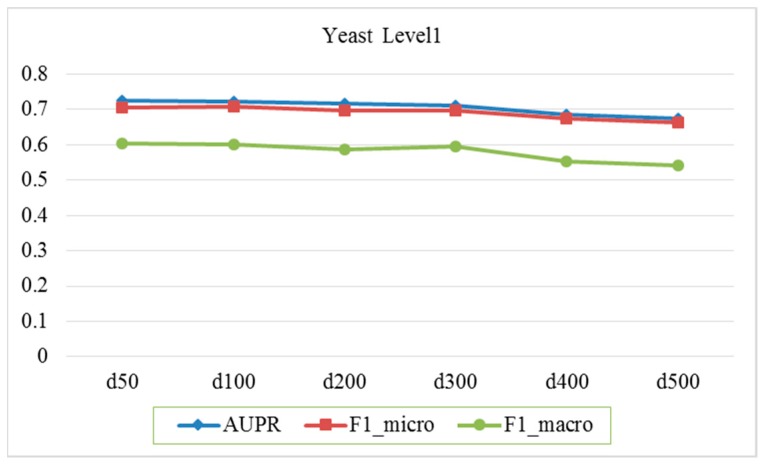
Performance comparison under different network feature dimensions.

In this paper, the topology features of each node (entity) in one network are extracted by running random walk algorithm on the network, but the obtained features tend to have higher dimensions and contain noise. For this reason, the diffusion component analysis (DCA) method is used to reduce the dimension [32,33]. In this section, the sensitivity of the feature dimension is discussed. Specifically, we evaluate how the feature dimension of each network affects the performance. In this experiment, 5-fold cross-validation is used to evaluate the effect of feature dimensions on performance based on yeast six protein interaction networks and functional labels of Level 1. We preset the random walk restart probability *a* = 0.5 and vary the dimension of the feature, setting the dimensions to 50, 100, 200, 300, 400, 500, etc. The predictive performance of the gene function is tested through Macro-averaged F1, Micro-averaged F1, and AUPR (the micro-averaged area under the precision–recall curve) metrics. As shown in Figure 1, the abscissa stands for the feature dimension of each network and the ordinate for the score. The predicted scores is the average of five trials.

As shown in the figure, when the dimension is increased from 50 to 500, the scores of metrics such as Macro-averaged *F*_1_, Micro-averaged *F*_1_, and AUPR do not change greatly. It is only when the dimension is greater than 300 that the corresponding score begins to slowly decline. In the experiments, the feature dimension of each network is set to 100.

### 2.4. Performance Evaluaton of Multinetwork Integration

**Figure 2:**
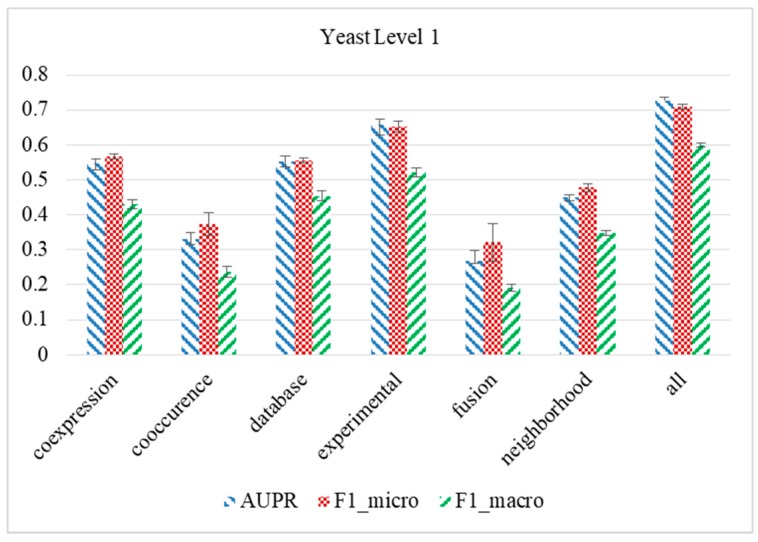
Comparison of predictive performance of multiple network integration with performance of single network.

An important factor that ReprsentConcat proposed in this paper can improve accuracy is the compactness of its feature representations, which not only helps to eliminate noise in the data, but also extracts functionally related topological patterns. In order to demonstrate the effectiveness of integrating multiple STRING networks, ReprsentConcat is applied to respectively single network in STRING, and the evaluation of function prediction for MIPS yeast annotations for Level 1 is performed. We compare the predictive performance on each individual network in STRING to using all networks simultaneously through 5-fold cross-validation. As shown in Figure 2, the cross-validation performance of ReprsentConcat is measured by metrics including Macro-averaged F1, Micro-averaged F1, and AUPR, as well as others. The results show that the prediction performance of all networks used at the same time (the bar with the horizontal axis of ‘all’ in the figure) is significantly better than the prediction performance of a single network (rank-sum test p value < 0.01). The results are summarized over five trials.

### 2.5. Comparison of Different Integrative Methods

**Figure 3:**
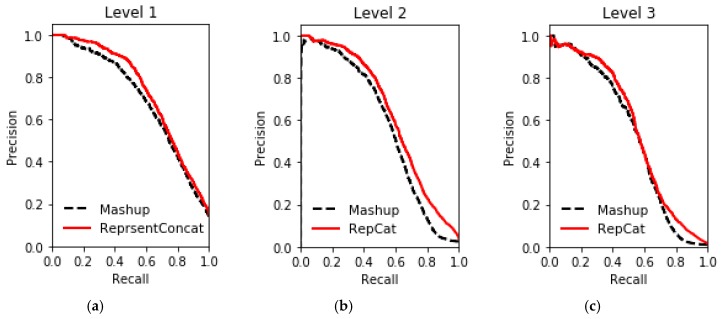
Comparison of performance on yeast datasets between ReprsentConcat and Mashup: (**a**) Level 1; (**b**) Level 2; and (**c**) Level 3.

**Figure 4:**
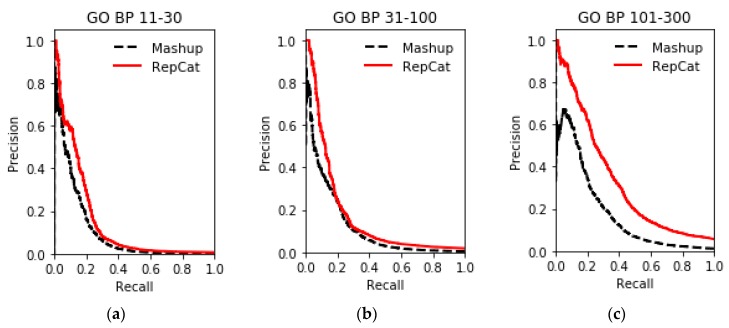
Performance Comparison of GO BP function prediction on human datasets between ReprsentConcat and Mashup. (**a**): GO BP 11-30; (**b**): GO BP 31-100; (**c**): GO BP 101-300.

**Figure 5:**
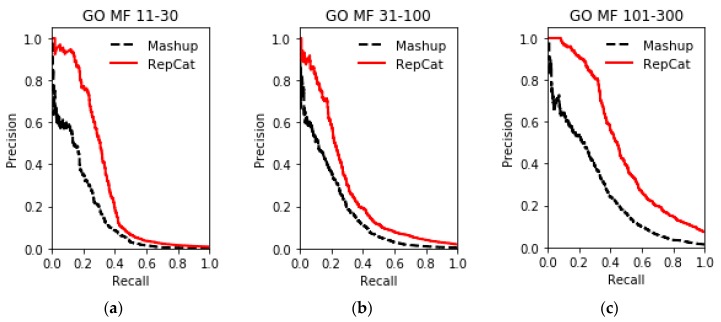
Performance comparison of GO MF function prediction on human datasets between ReprsentConcat and Mashup: (**a**) GO MF 11-30; (**b**) GO MF 31-100; and (**c**) GO MF 101-300.

**Figure 6:**
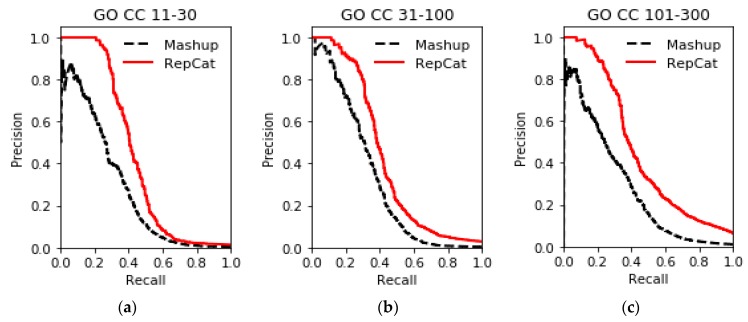
Performance comparison of GO CC function prediction on human datasets between ReprsentConcat and Mashup: (**a**) GO CC 11-30; (**b**) GO CC 31-100; and (**c**) GO CC 101-300.

**Figure 7:**
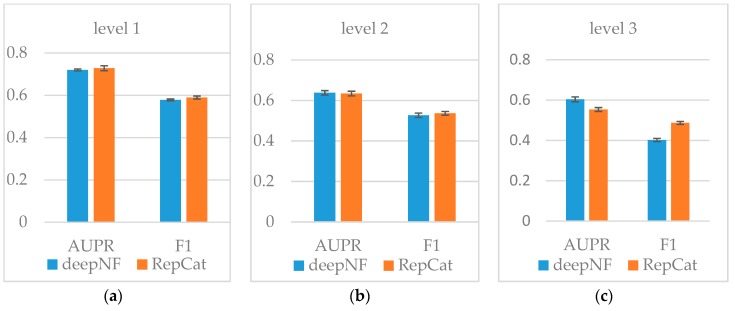
Performance comparison on yeast dataset between ReprsentConcat and deepNF: (**a**) Level 1; (**b**) Level 2; and (**c**) Level 3.

The results of gene function prediction on multiple networks in the STRING database using ReprsentConcat are shown in Figure 3, Figure 4, Figure 5, Figure 6 and Figure 7. In the ReprsentConcat method, the restart probability, which is a parameter in random walk algorithm, is set to 0.5. We also experimentally confirm that the performance of ReprsentConcat is stable when the restart probability varies between 0.1 and 0.9. Due to the different protein interaction networks between yeast and humans, different dimensions are chosen when reducing the dimension of network topology features. For six yeast proteins interaction networks, the dimension is 100, and for human protein interaction networks, the dimension is 300. In the experiment, we employ gcForest for multinetwork integration and function prediction. Each level in the cascade uses eight random forest classifiers, and each forest contains 500 trees. In order to automatically determine the optimal number of cascade levels, it is especially important to select appropriate evaluation metric. Considering that gene function prediction belongs to multilabel classification problem, we use *F*_1_ metric to determine the number of cascade levels. That is, if the prediction performance in the next four levels is not improved then, the current level is considered to be the optimal number of level, and the output of the current level is the final prediction result.

To evaluate the performance, ReprsentConcat is compared to the latest multinetwork integration methods: Mashup [27] and deepNF [28]. In the Mashup method, the high-dimension topological features of each node in the network were first obtained by random walk. When reducing the dimension of the high-dimension feature, it was assumed that the low-dimension features of the nodes in multiple networks were the same. Then the same low-dimension topology features of multiple networks were obtained by solving an optimization function. As shown in Figure 3, Figure 4, Figure 5 and Figure 6, according to the PR-curve, the ReprsentConcat (denoted as RepCat) method is superior to the Mashup method in the cross-validation experiment of gene function prediction in the real data sets of yeast and human. We demonstrate that ReprsentConcat has significant performance improvements at the different annotation levels of the MIPS database and the GO database. For example, in the function annotation MIPS Level 1, the AUPR values of Mashup and ReprsentConcat are 0.70 and 0.728, respectively. Part of the reason for the improved performance of ReprsentConcat is that it obtains the topology pattern of each network and compacts the representation of topological features. The compressed low-dimension feature helps to eliminate noise in the network, while gcForest based on random forests does the feature selection.

deepNF integrated different heterogeneous networks of protein interactions and extracted the compact, low-dimensional feature representation for each node by using the stack denoising autoencoder, then fed the representations into SVM classifiers. The method was able to capture nonlinear information contained in large-scale biological networks and the experiments indicated that it had a good performance on human and yeast STRING networks. We compare ReprsentConcat and deepNF by running 5-fold cross-validation on yeast STRING networks. The results on different annotation levels of the MIPS hierarchy are summarized in Figure 7 (ReprsentConcat denoted as RepCat). We observe that the two methods share similar performance regarding the AUPR and *F*_1_ at levels 1 and 2 of the MIPS hierarchy. At level 3, the AUPR value of deepNF is larger than that of ReprsentConcat while the *F*_1_ value of ReprsentConcat is larger. Since deepNF is based on deep neural networks, there are a number of hyperparameters (e.g., hidden layers, nodes in the hidden layer, and learning rate) to tune and the procedure generally is difficult and needs tricks and expertise. Moreover, the computational cost is usually high. In DeepNF, there are more than three hundred million parameters in the yeast networks to be trained in total. The training consumes almost all of the memory of the GPU (two Geforce RTX 2080 GPUs with 22GB memory in our server). Relatively few hyperparameters (the number of forests and trees in each forest) need to be set in ReprsentConcat, and the training can be performed on CPU.

### 2.6. Case Study: ESR1

Estrogen signaling is mediated by binding to estrogen receptors (ERs), which are ligand-dependent transcription factors composed of several domains important for hormone binding, DNA binding, and activation of transcription. There exist two ER subtypes in humans, namely ERα and ERβ, coded by the *ESR1* and *ESR2* genes, respectively [34]. Gene *ESR1* is located on chromosome 6q25.1 and consists of eight exons spanning >140 kb. The protein coded by *ESR1* localizes to the nucleus where it may form a homodimer or a heterodimer with estrogen receptor 2. The researches have demonstrated that estrogen and its receptors are essential for sexual development and reproductive function, but are also involved in other tissues such as bone. Estrogen receptors are also involved in pathological processes including breast cancer, endometrial cancer, and osteoporosis [35,36]. There is strong evidence for a relationship between genetic variants on the ESR1 gene and cognitive outcomes. The relationships between ESR1 and cognitive impairment tend to be specific to or driven by women and restricted to risk for Alzheimer’s disease rather than other dementia causes [37]. 

We employ ReprsentConcat to predict the functions of gene ESR1. As described above, the GO terms, which are divided into three categories (namely, BP, MF, and CC), which are further split into three groups for each category according to the number of annotated genes. In the category of BP, there are 28 GO terms with 101–300 annotated genes. In this experiment, we predict the functions of ESR1 by using the protein interaction networks and the 28 GO labels. The output of ReprsentConcat is a 28-dimensional probability vector in which each entry represents the probability of having the function. The vector is sorted and the result is listed in Table 3. The GO terms marked with the character ‘#’, which have been confirmed in our annotation datasets, are ranked 2nd and 16th, respectively. The GO terms marked with character ‘*’, which are new annotations and confirmed in 2017 from UniProt-GOA [38], ranked 1st, 4th, 9th, 10th, and 15th, respectively. The result shows ReprsentConcat generates relatively satisfactory predictions.

## 3. Multinetwork Integration Based on gcForest

### 3.1. gcForest

Ensemble learning has been well studied and widely deployed in many applications [39,40,41,42,43]. As described in Section 1, gcForest is an ensemble method based on forest. Its structure mainly includes cascade forest and multigrained scanning.

#### 3.1.1. Cascade Forest

**Figure 8:**
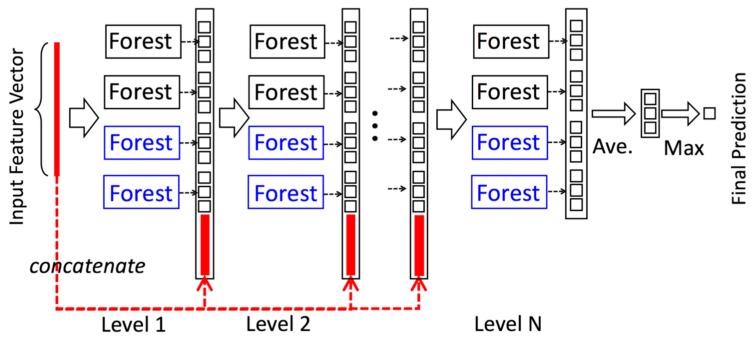
The cascade structure of gcForest.

gcForest’s cascade structure adapts a level after level structure of deep network, that is, each level in the cascade structure receives the processed result of the preceding level, and passes the processed result of the level to the next level, as shown in Figure 8. Each level is composed of multiple random forests made up of decision trees. In Figure 8, there are two random forests, which are completely random forest (black) and random forest (blue), respectively.

Each forest will generate a probability vector of length *C*. If each level of gcForest is composed of *N* forests, then the output of each level is *N C*-dimensional vectors connected together, namely, C*N dimensional vectors. The vector is then spliced with the original feature vector of the next level (the thick red line portion of each level in Figure 8) as the input to the next level. For example, in the three-classification problem in Figure 8, each level consists of four random forests, and each forest will generate a 3-dimensional vector. Hence, each level produces a 4*3=12-dimensional feature vector. This feature vector will be used as augmented feature of the original feature for the next level. To reduce the risk of overfitting, the class vector generated in each forest is produced by k-fold cross-validation. Specifically, after extending a new level, the performance of the entire cascade will be evaluated on the validation set, and the training process will terminate if there is no significant performance improvement. Therefore, the number of cascade levels in cascade is automatically determined.

#### 3.1.2. Multigrained Scanning

Since there may be some relationships between the features of the data, for example, in image recognition, there is a strong spatial relationship between pixels close in position, and sequential relationships between sequence data. Cascade forest is enhanced through multigrained scanning, i.e., it samples by sliding windows with a variety of sizes to obtain more feature subsamples, so as to achieve the effect of multigrained scanning. 

**Figure 9:**
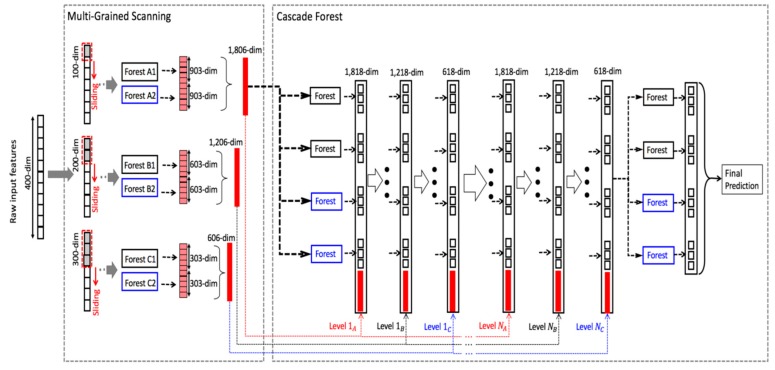
The overall structure of gcForest.

By employing multiple sizes of sliding windows, the final transformed feature vector will include more features, as shown in Figure 9. In Figure 9, it is assumed that the 100-dimensional, 200-dimensional, and 300-dimensional windows are used to slide on the raw 400-dimensional features.

### 3.2. Network Feature Extraction

The method of random walk with restart (RWR) has been widely used in network structure analysis [44,45,46,47,48]. The RWR algorithm allows the restart of a random walk from the initial node at each step with a certain probability. It can capture local and global topology information to identify important nodes in the network. Assuming that a protein interaction network containing *n* nodes is represented by *G* = (*V*, *E*), where *V* is the set of nodes, each node representing a protein, and *E* is the set of edges. *A* is the adjacency matrix of *G*. *M* represents the Markov possibility transition matrix of *A*, and each element *M_ij_* denotes the probability walking from node *j* to node *i*, then,

(1) $$M_{ij}=\frac{A_{ij}}{\sum_{i\prime }A_{i\prime j}}$$

The iterative equation for the random walk from node *i* is as follows,

(2) $$s_{i}^{t+1}=(1-\alpha )s_{i}^{t}M+\alpha s_{i}^{0}$$

a is the restart probability, which determines the relative importance of local and global topology information. The larger its value, the greater the chances of restart, and the more important the local structure information. *s_i_* is an distribution vector of *n*-dimension, where each entry represents the probability that a node is visited after *t*-walk; $s_{i}^{0}$ denotes the initial probability, and $s_{i}^{0}(i)=1$, $s_{i}^{0}(j)=0$. After several iterations, *s_i_* can converge to a stable distribution, then this distribution represents the probability of a transition from node *i* to node *j*, including the topological information of the path from node *i* to node *j*. Then, if there are similar diffusion states between node *i* and node *j*, it means that they have similar positions in the network, which implies that they might have similar functions. Hence, when the RWR is stable, we obtain the diffusion state feature of each node.

The feature dimension obtained by random walk is high. We use diffusion component analysis (DCA) [27] to reduce the dimension. To extract a fewer dimensional vector representation of nodes, we employ the logistic model to approximate diffusion state *s_i_* of each node. In detail, the probability of random walk from node *i* to node *j* is specified by

(3) $${\hat{s}}_{ij}=\frac{\exp\{x_{i}^{T}w_{j}\}}{\sum_{j\prime }\exp\{x_{i}^{T}w_{j\prime }\}}$$

Where *x*_i_ and *w_j_* are *d*-dimension vectors and *d* is much smaller than *n*. *x_i_* represents the node features, and *w_i_* represents the context features, both of which capture the topology information of the network. The inner product is larger when the *x_i_* and *w_j_* are closer in direction, which implies that random walks starting from node *i* will frequently visit node *j*. In order to calculate *w* and *x*, we define the KL-divergence distance between the real distribution *s_i_* and the transformed distribution ${\hat{s}}_{i}$ and minimize it, namely, the loss function for *n* nodes is

(4) $$\underset{w,x}{\min}C(s,\hat{s})=\frac{1}{n}\sum_{i=1}^{n}D_{KL}(s_{i}||{\hat{s}}_{i}).$$

We can obtain the low-dimensional feature by solving the minimum value of this loss function

### 3.3. Training and Prediction of ReprsentConcat

In ReprsentConcat, the *d*-dimension topology features of each network are first obtained according to the method described above, and then the topological features of multiple networks are concatenated to generate a one-dimension feature vector as the input features of gcForest. Considering that there is no spatial or sequential relationship between these features, we do not perform the multigrained process on these features. In the training, the prediction performance of each level is evaluated by *k*-fold cross-validation. We use Micro-averaged *F*_1_ as the metric to determine the number of cascade levels. The outputs of the current level are considered to be the final predictions if there is no improvement in the next *m* levels in term of *F*_1_. The pseudocode of ReprsentConcat is shown in Algorithm 1.


**Algorithm 1: ReprsentConcat Algorithm**
**Input**: *network_files*: paths to adjacency list files, *n*: number of genes in input networks, *d*: number of output dimensions, *onttype*: which type of annotations to use, *early_stopping_rounds*: number of stopping the rounds **Output**: *opt_pred_results*: prediction results **for***i=1*: length( *network_files*)  *A*=load_network( *network_files*(i), *n*)  *Q*=rwr(*A*, 0.5)   *R*=ln(*Q*+1/*n*)  *U*, ∑, *V* =svd(*R*)  $X\_cur=U_{d}\sum_{d}^{1/2}$  *X*=hstack(*X*, *X_cur*) **end for** *Y*=load_annotation(*onttype*) //load annotations //split the data into train data and test data *X_train*, *Y_train*, *X_test*, *Y_test*=train_test_split(*X*, *Y*)  *layer_id*=0 **while** 1  **if**
*layer_id*==0   *X_cur_train*=zeros(*X_train*)   *X_cur_test*=zeros( *X_test*)  **else**   *X_cur_train*=*X_proba_train*.copy()   *X_cur_test*= *X_proba_test*.copy()  **end if**  *X_cur_train*=hstack( *X_cur_train, X_train*)  *X_cur_ test* =hstack( *X_cur_ test, X_ test*)  **for**
*estimator*
**in**
*n_randomForests*   //train each forest through k-fold cross validation   *y_probas*= *estimator.*fit_transform( *X_cur_train, Y_train*)   *y_train_proba_li*+= *y_probas*   *y_test_probas*= *estimator.predict_proba*(*X_cur_ test*)   *y_test_proba_li*+= *y_test_probas*  **end for**  *y_train_proba_li* /=length(*n_randomForests*)  *y_test_proba_li* /=length(*n_randomForests*)  *train_avg_F_1_*=calc_F1(*Y_train, y_train_proba_li*) // calculate the F_1_ value  *test_avg_F_1_*=calc_F1(*Y_test, y_test_proba_li*)  *test_F_1__list*.append( *test_avg_F_1_*)  *opt_layer_id*=get_opt_layer_id( *test_F_1__list*)  **if**
*opt_layer_id* = layer_id   *opt_pred_results*=[ *y_train_proba_li, y_test_proba_li*]  **end if**  **if**
*layer_id* - *opt_layer_id* >= *early_stopping_rounds*   **return**
*opt_pred_results*  **end if**  *layer_id*+=1 **end while**

In order to obtain the predictions in a test set, the features of a test sample are fed to the cascade forest. The output of the optimal level which is determined by the training process is a multidimensional class vector. Each entry of the class vector is a probability indicating the possibility that the sample belongs to one class. Hence, a threshold *t* is applied to the class vector to obtain predictions for all classes. If the *j*th value of the class vector is equal to or larger than the given threshold, the sample is assigned to the class *C^j^* where *C* represents the set of classes. The final classification result of ReprsentConcat is given by a binary vector *V* with the length of |*C*|. If the *j*th output is equal to or larger than the given threshold, *V_j_* is set to 1. Otherwise, it is set to 0. Obviously, different thresholds may result in different predictions. Since the output of cascade forest is between 0 and 1, the thresholds also vary between 0 and 1. The larger the threshold used, the less the predicted classes. Conversely, the smaller the threshold used, the more the predicted classes.

## 4. Conclusions

In this paper, we propose ReprsentConcat, an integrative method, to combine multiple networks from heterogeneous data sources. In ReprsentConcat, the topological features are extracted by running random walks on each network, and the features are represented using low-dimensional vectors. Then the low-dimensional features are concatenated as the input of gcForests for prediction. To verify the performance of this method, we performed gene function prediction on multiple protein interaction networks of yeast and humans. The experimental results demonstrated that the prediction performance by integrating multiple networks is much better than that using a single network. Moreover, ReprsentConcat is not sensitive to multiple parameters such as the number of dimensions for function prediction. We also compare with the latest network integration method Mashup. According to the result of 5-fold cross-validation, ReprsentConcat outperforms Mashup in terms of precision–recall curves.

Besides the network data, other non-network information, such as sequence features, can be integrated into ReprsentConcat for function prediction by concatenating them. As a note, there are still further improvements in the predictions of protein function in our method. For example, the topological features of nodes are extracted through semisupervised learning by combining label information. As a result, the learned features might be more effective in this manner.

## Figures and Tables

**Table 1:** Interaction network and its corresponding number of edges.

Network	Human	Yeast
coexpression	788,166	314,014
co-occurrence	18,064	2664
database	159,502	33,486
experimental	309,287	219,995
fusion	1880	1361
neighborhood	52,479	45,610

**Table 2:** Number of Gene Ontology (GO) terms by the number of annotated genes in human biological process (BP)/molecular function (MF)/cellular component (CC).

	11–30	31–100	101–300
BP	262	100	28
MF	153	72	18
CC	82	46	18

**Table 3:** The rank of GO terms according the predictions of ReprsentConcat. The GO terms marked with the character ‘#’ indicate that they have been confirmed in the annotation datasets, and the GO terms marked with the character ‘*’ represent they are new annotations for 2017 from UniProt-GOA.

Rank	GO Term	GO Name
1	GO:0000122 *	negative regulation of transcription by RNA polymerase II
2	GO:0071495 #	cellular response to endogenous stimulus
3	GO:0016265	obsolete death
4	GO:0048878*	chemical homeostasis
5	GO:0051241	negative regulation of multicellular organismal process
6	GO:0051098	regulation of binding
7	GO:0008284	positive regulation of cell population proliferation
8	GO:0007399	nervous system development
9	GO:0006259*	DNA metabolic process
10	GO:0009057*	macromolecule catabolic process
11	GO:0010564	regulation of cell cycle process
12	GO:0043900	regulation of multi-organism process
13	GO:0002520	immune system development
14	GO:0006928	movement of cell or subcellular component
15	GO:0006325*	chromatin organization
16	GO:0018130#	heterocycle biosynthetic process
17	GO:0016192	vesicle-mediated transport
18	GO:0031647	regulation of protein stability
19	GO:0003008	system process
20	GO:0008283	cell population proliferation
21	GO:0051259	protein complex oligomerization
22	GO:0030111	regulation of Wnt signaling pathway
23	GO:0006629	lipid metabolic process
24	GO:0034622	cellular protein-containing complex assembly
25	GO:0010608	posttranscriptional regulation of gene expression
26	GO:0055085	transmembrane transport
27	GO:0016311	dephosphorylation
28	GO:0007186	G protein-coupled receptor signaling pathway
